# Magnetic photonic crystals for biomedical applications

**DOI:** 10.1002/SMMD.20220039

**Published:** 2023-03-20

**Authors:** Hanxu Chen, Ning Li, Zhuxiao Gu, Hongcheng Gu, Jinglin Wang

**Affiliations:** ^1^ Department of Hepatobiliary Surgery Nanjing Drum Tower Hospital School of Biological Science and Medical Engineering Southeast University Nanjing China

**Keywords:** biomedical, bottom‐up, magnetic, photonic crystal, self‐assembly

## Abstract

Magnetic photonic crystals (PhCs), as a representative responsive structural color material, have attracted increasing research focus due to merits such as brilliant refraction colors, instant responsiveness, and excellent manipuility, thus having been widely applied for color displaying, three‐dimensional printing, sensing, and so on. Featured with traits such as contactless manner, flexible orientations, and adjustable intensity of external magnetism, magnetic PhCs have shown great superiority especially in the field of biomedical applications such as bioimaging and auxiliary clinical diagnosis. In this review, we summarize the current advancements of magnetic PhCs. We first introduce the fundamental principles and typical characteristics of PhCs. Afterward, we present several typical self‐assembly strategies with their frontiers in practical applications. Finally, we analyze the current situations of magnetic PhCs and put forward the prospective challenges and future development directions.

## Graphical abstract

Magnetic photonic crystals have been regarded as a representative responsive structural color material. Benefitting from merits such as brilliant refraction colors, instant responsiveness, and excellent manipuility, such materials have been widely applied for color displaying, three‐dimensional printing, sensing, and so on. Featured with traits such as contactless manner, flexible orientations, and adjustable intensity of external magnetism, magnetic photonic crystals have shown great superiority, especially in the field of biomedical applications such as bioimaging and auxiliary clinical diagnosis. Herein, the fundamental principles and the current advancements of magnetic photonic crystals are first summarized. Afterward, we present several typical self‐assembly strategies with their frontiers in practical applications. Finally, we analyze the current situations of magnetic photonic crystals and put forward the prospective challenges and future development directions.

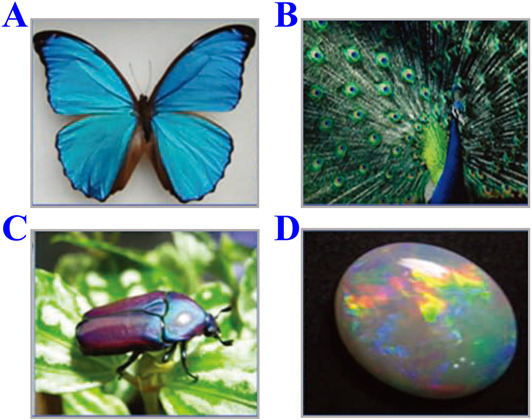

## 1 INTRODUCTION

**FIGURE 1:**
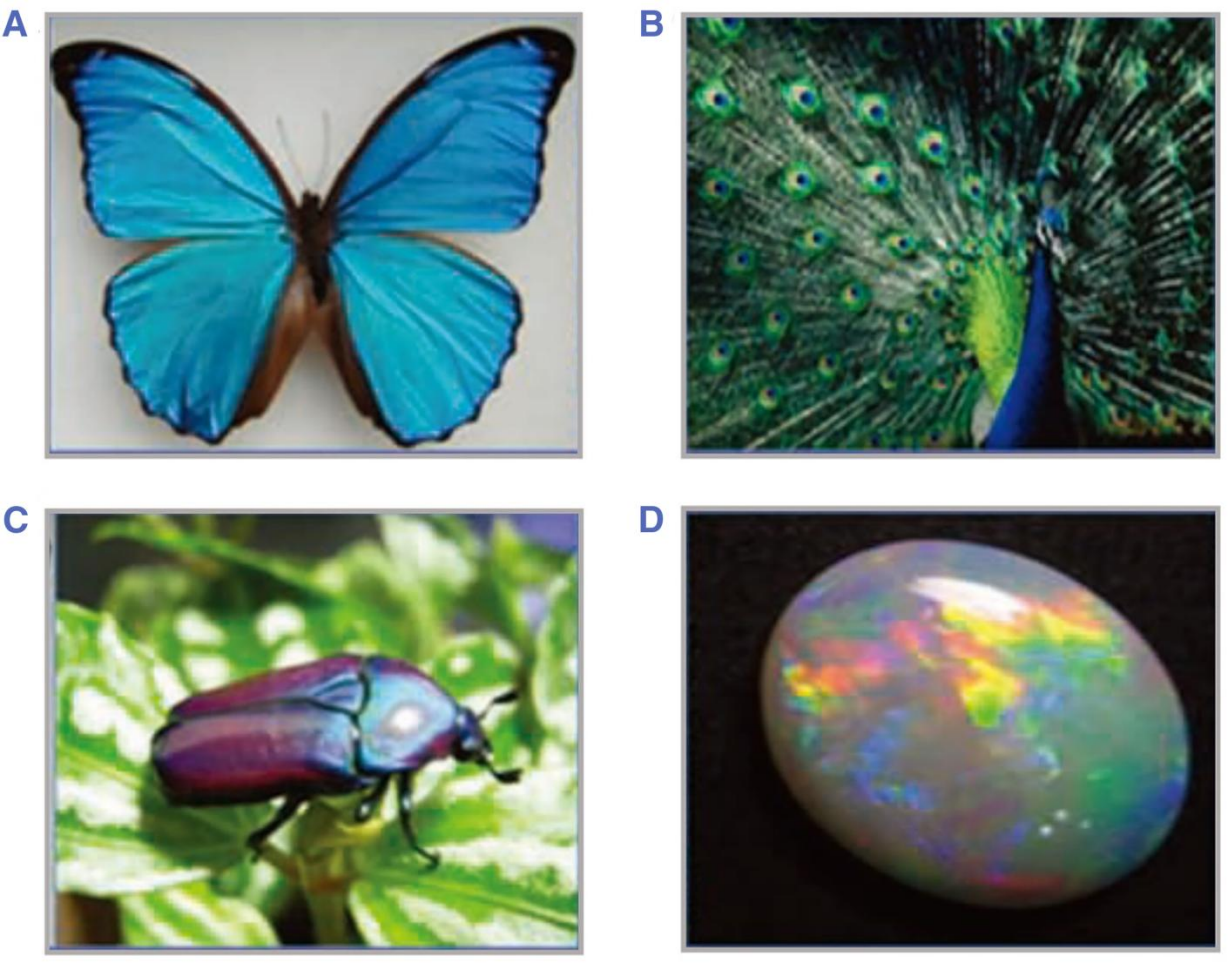
The photonic crystals observed on natural liver species. (A) Morpho Menelaus. (B) Peacock. (C) Jewel beetle. (D) Opals. Reproduced with permission.^7^ Copyright 2010, WILEY‐VCH Verlag GmbH & Co. KGaA, Weinheim.

Photonic crystal (PhCs) materials have been widely observed and explored in abundant natural living species, such as butterfly wings, feathers of certain birds, fish skins, insects, etc.^1^, ^2^, ^3^, ^4^, ^5^, ^6^, ^7^ Inherently periodically ordered spatial arrangements of the dielectric PhC materials enable the manipulation of the lights within characteristic photonic band gaps (PBGs) and appear as brilliant structural colors, as presented in Figure 1.

**Key points**

- The traditional preparation strategies of magnetic photonic crystals (PhCs) are comprehensive summarized.
- Diverse materials choices are proposed for fabricating magnetic PhCs.
- The novel practical biomedical applications of magnetic PhCs are reviewed and future development directions are presented.

Inspired by such periodic architectures (one‐, two‐, and three‐dimension), numerous technologies (e.g., lithography, etching, chemical deposition, and self‐assembly) have been explored to better construct artificial PhC materials with flexible and enriched properties, especially responsiveness to external environmental stimuli, namely responsive PhCs.^8^, ^9^, ^10^, ^11^, ^12^, ^13^, ^14^, ^15^ By performing stimuli such as temperature, light, and acoustic, electrical, and magnetic fields, such materials are imparted with tunable PBG properties (diffractive wavelength or intensity) and applied to a wider range of practical applications, including reflective coatings, specific waveguides, coloration encoding, and so on.^16^, ^17^, ^18^, ^19^, ^20^, ^21^, ^22^, ^23^, ^24^

Among these types of responsive PhCs, magnetic PhCs are receiving increasing attention owing to the wider integration of magnetic approaches within the field of biomedical applications.^25^, ^26^, ^27^, ^28^, ^29^, ^30^, ^31^, ^32^ In addition, evolved with the distinctive traits of contactless manner, instantaneous response, flexible orientations, and adjustable intensity of external magnetic field, magnetic PhCs can achieve breakthroughs in the more convenient manipulation and diversified functions, such as bioimaging, biosensing, and auxiliary clinical diagnosis.^23^, ^24^, ^25^, ^26^, ^27^, ^28^, ^29^, ^30^, ^31^, ^32^, ^33^, ^34^, ^35^, ^36^, ^37^ In this review, we present a summary of the advanced progress of magnetic PhCs in practical applications. We begin with the several main adopted magnetic assembly mechanisms. Then, we emphasize their frontiers applications in the biomedical field. Finally, we sum up the current status and look at the remaining challenges with future directions on the basis of the achievements.

## 2 FABRICATION OF MAGNETIC PhCs

### 2.1 Fundamental of PhCs

**FIGURE 2:**
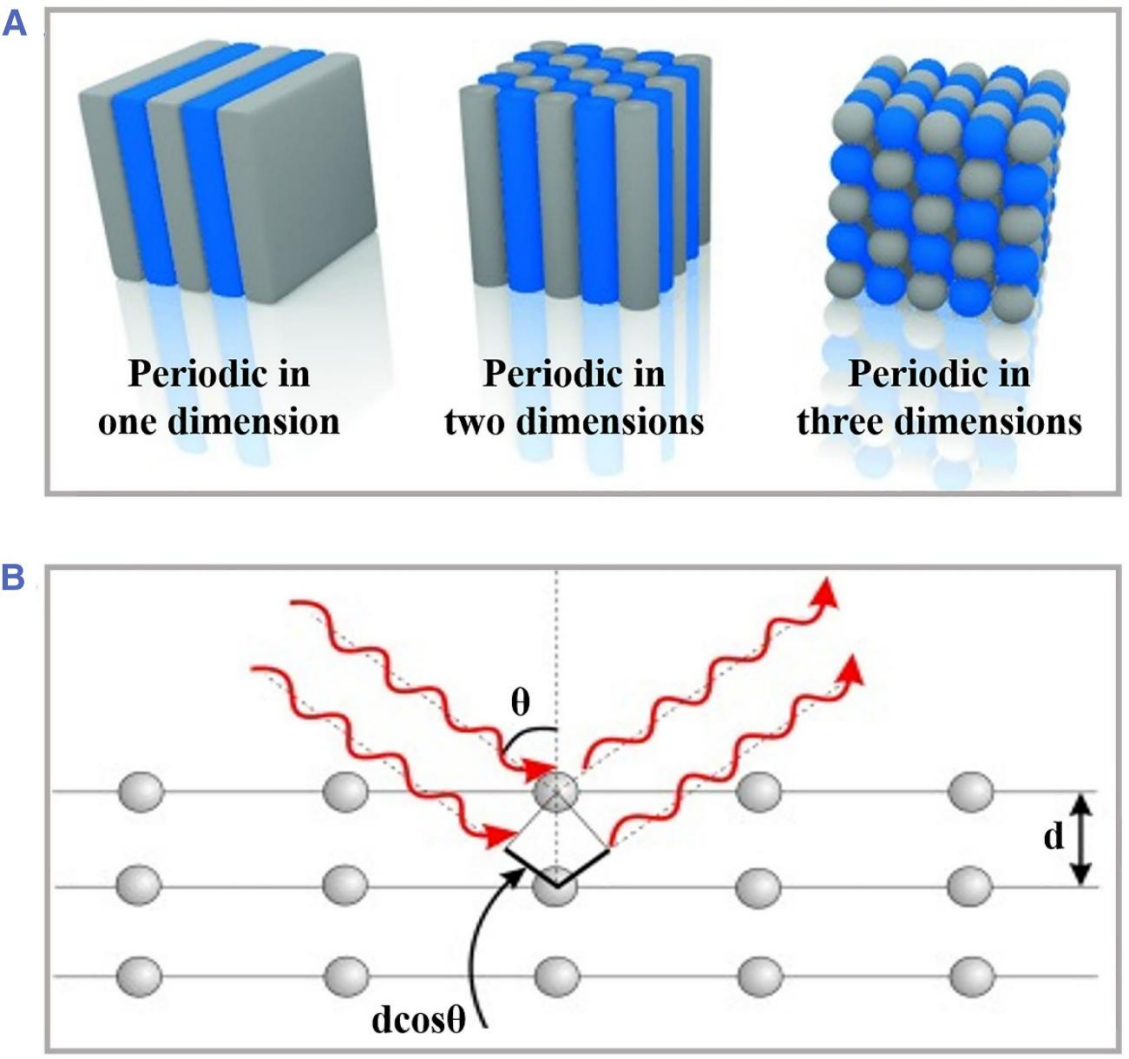
Fundamental of PhCs. (A) Schematic representation of the three kinds of PhCs. (B) Mechanism of reflection of light with specific wavelength within periodically ordered structures. Reproduced with permission.^47^ Copyright 2014, WILEY‐VCH Verlag GmbH & Co. KGaA, Weinheim. PhCs, photonic crystals.

Recently, diverse methods have been proposed to replicate the periodic photonic structures of PhCs, which can be roughly divided into two classifications: top‐down and bottom‐up strategy. The top‐down strategy mainly relies on traditional microfabrication techniques such as lithography, etching, chemical evaporation, and deposition, which directly manufacture micro/nanostructures with the aid of high‐precision computer‐assisted instruments.^38^, ^39^, ^40^, ^41^, ^42^, ^43^, ^44^, ^45^, ^46^ However, such a manner has been restricted due to the high cost, complex operations, and limited nanoscale precision. In contract, the bottom‐up strategy, mainly referred as self‐assembly, could achieve synthesis of hierarchical highly‐ordered PhC architectures (one‐, two‐, and three‐dimension) with low cost, high efficiency, and delicate nanoscales, as schemed in Figure 2A. Besides, the procedures of self‐assembly could be easily customized to satisfy rigorous requirements. Hence, the self‐assembly strategy has been widely adopted to fabricate diverse one‐, two‐, and three‐dimensional PhC materials by equilibrium phase segregation of well‐defined block copolymers or monodispersed colloidal nanoparticles (e.g., silica oxide, titania oxide, polystyrene, polyacrylates, and ferroferric oxide).^48^, ^49^, ^50^, ^51^

As mentioned above, PhC materials appear as iridescent structural colors due to the periodic nanostructures‐derived PBG, where only light of certain wavelengths could be reflected (Figure 2B).^47^, ^52^, ^53^ The characteristic wavelength of PhCs could be calculated according to the following Bragg's equation:

2dcosθ=mλ

where d is the distance between neighboring colloidal particles, θ is the angle of incident light, m refers to the diffraction order, and λ is the wavelength. Considering the influence caused by the surrounding medium around the PhCs, the above equation could be integrated with Snell's law and deduced as another format:

2dneff2−sin2θ12=mλ

$$n_{eff}^{2}=n_{p}^{2}V_{p}+n_{m}^{2}V_{m}$$

where $n_{eff}$ is the average index of refraction of the monolithic PhCs, $n_{p}$ and $n_{m}$ refer to the refractive index of particles and surrounding medium, and $V_{p}$ and $V_{m}$ refer to the respective volume fraction. According to this equation, it is found that the position of λ is mainly determined by the distance of between neighboring particles and average refractive index. The former parameter could be regulated by optimizing reaction conditions including pH values, charge polarity, chemical interactions, or special confined locations during the assembly process. As to the latter parameter, functional materials bearing a tunable refractive index could serve as medium components to realize dynamic variable optical properties of PhCs.^54^, ^55^, ^56^, ^57^, ^58^, ^59^, ^60^, ^61^ Typically, by introducing stimuli‐responsive copolymers into the skeleton, the resultant responsive PhCs could appear as corresponding variations of optical properties under external environmental stimuli, including physical and chemical stimuli. In brief, the external manipulations on self‐assembly process could impart resultant PhCs with abundant individual properties for wider applications.

### 2.2 Magnetic assembly of PhCs

As we reviewed above, the self‐assembly process could be integrated with chemical or physical interventions to endow PhCs with more interesting properties. Among these methods, external magnetism has emerged as a powerful tool owing to merits such as contactless manner, flexible orientations, immediate response, and tunable intensity. Generally, the magnetic assembly of colloidal nanoparticles toward PhCs can be classified into three types, including 1D chain‐like structures, 2D planar structures, and 3D colloidal crystals, depending on the final morphology.^21^, ^62^, ^63^, ^64^, ^65^, ^66^, ^67^, ^68^

**FIGURE 3:**
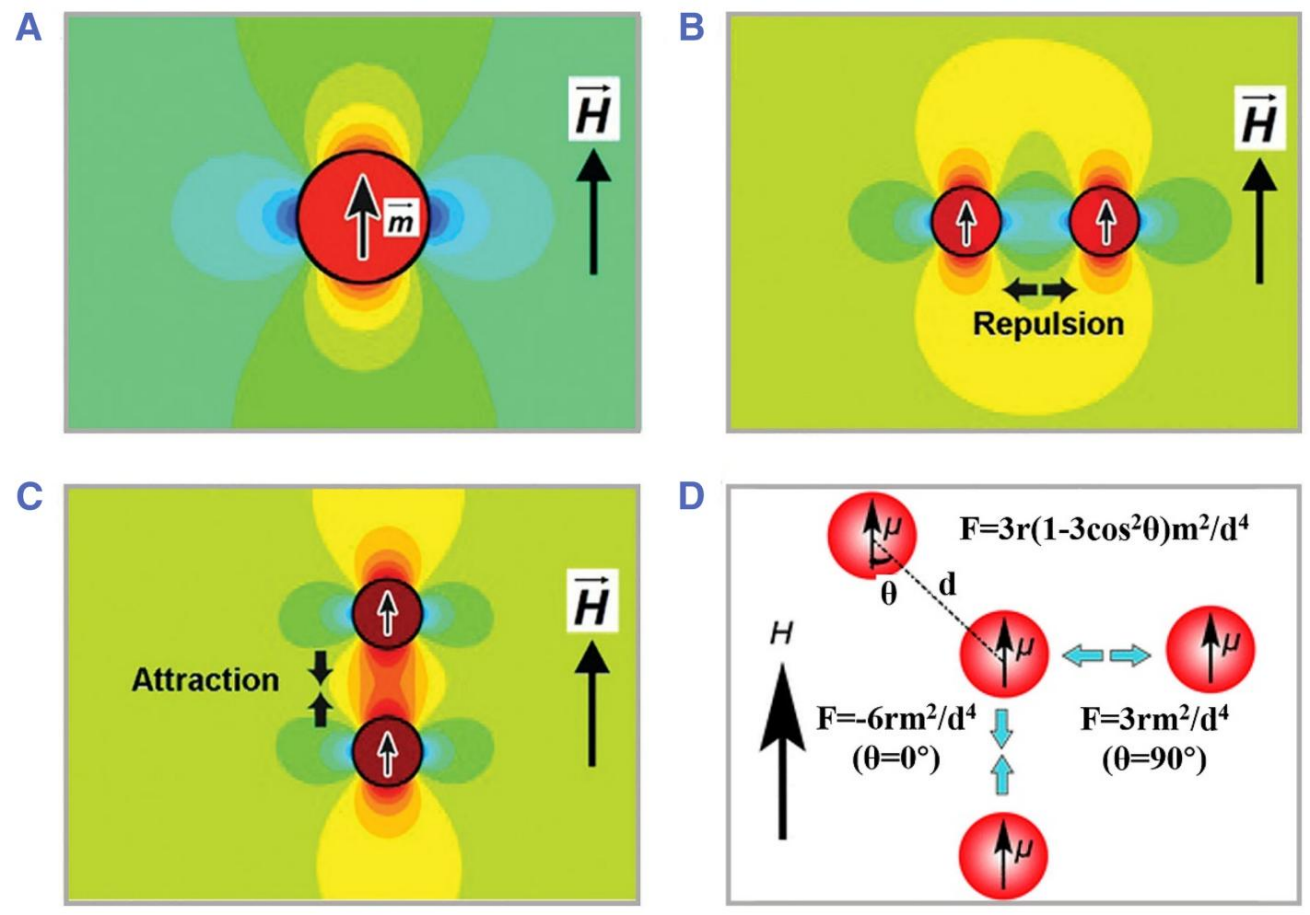
Principles of magnetite nanoparticles in external magnetic fields. (A) Magnetic field distribution around a superparamagnetic particle with a dipole moment. (B) Repulsive force in horizontal configuration. (C) Attractive force in vertical configuration. Reproduced with permission.^69^ Copyright 2012, American Chemical Society. (D) Scheme of the dipole–dipole interactions between two neighboring nanoparticles. Reproduced with permission.^70^ Copyright 2011, Royal Society of Chemistry.

When applying an external magnetic field, colloidal particles would be immediately magnetized with an induced magnetic dipole moment; thus, this particle produces its own magnetic field and exerts forces on adjacent particles (Figure 3A–C).^69^, ^71^ The dipole–dipole force exerted by the particles could be calculated as the following equation:

$$F_{21}=3r\left(1-3\;\cos^{2}\;\theta \right)m^{2}/d^{4}$$

where r is the unit vector pointing from the particle to the adjacent one, θ is the angle between the direction of r and field direction, m is magnetic moment of the magnetized particle, and d is the distance between two particles. According to the above expression, the dipole–dipole force is mainly determined by the configuration of two neighboring particles. It could be calculated that the force appears attractive when θ is smaller than 54.09° and repulsive when θ is ranging from 54.09° to 90°, as presented in Figure 3D.^70^ Definitely, the particles within the magnetic field experience another force, namely packing force. Such a packing force is due to the heterogeneity of the experimental magnetic field and drives the particle along the direction of the maximum magnetic field strength. Based on the optimization of the equilibration balance between dipole–dipole forces, packing forces, long‐range interparticle repulsions, and thermal fluctuations, colloidal particles could be assembled into different morphologies. Different from traditional natural self‐assembly mechanism where the functional units assemble themselves due to interactions, solvent evaporation, or solvent replacement, magnetic self‐assembly processes mainly depend on the assistance of external magnetism and respective magnetic responsiveness of inner nanoparticles/nanoclusters.

**FIGURE 4:**
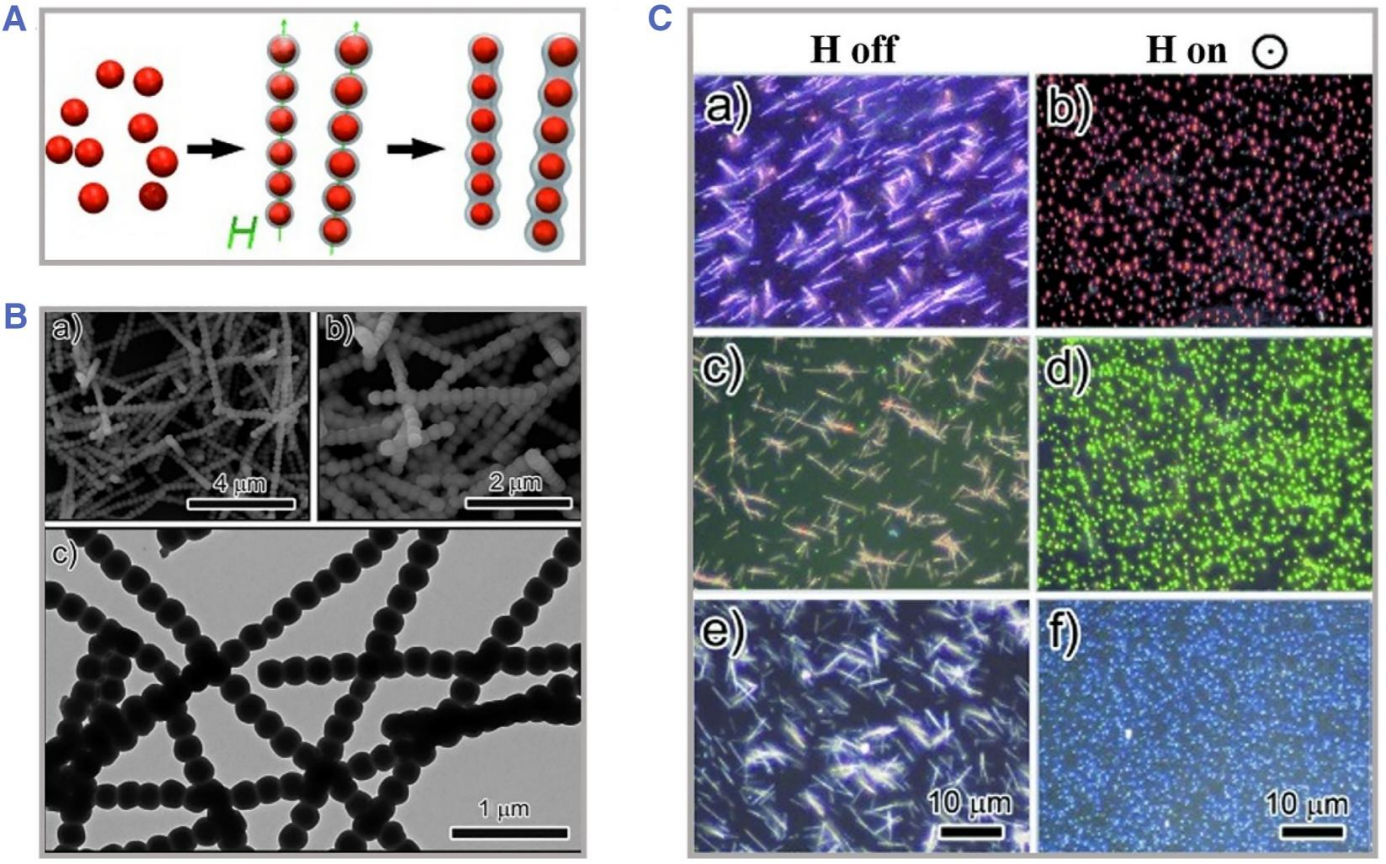
The assembly of 1D‐chain magnetic photonic crystals. (A) Scheme of the fabrication of nanoparticles distributed randomly toward chain‐like structures under external magnetic field. (B) Electron microscopy images of 1D photonic chains. (C) Optical images of 1D photonic chains with and without an external magnetic field. Reproduced with permission.^78^ Copyright 2011, WILEY‐VCH Verlag GmbH & Co. KGaA, Weinheim.

The dipole–dipole force serves as a critical role of forming 1D chain‐like structures. When the dipole–dipole force is strong enough to overcome the thermal fluctuations, the colloidal particles tend to be aligned along the magnetic moments' directions. The electrostatic repulsive forces are balanced with dipole–dipole attractive forces if the size of particles is extremely homogeneous. Meanwhile, the dipole–dipole repulsive forces between neighboring chains could also separate single chains from each other. By slightly adjusting the intensity or the orientation of the magnetic field, the interparticle spacing and structural periodicity could be easily altered. Besides, benefitting from the rapid magnetization rates, this assembly process tends to be instant.^72^, ^73^, ^74^, ^75^ The optical properties of 1D colloidal chain would respectively change and the characteristic wavelength could be deduced according to above Bragg's expression, which enables 1D magnetic PhCs for rapid and sensitive measurements. In addition, the diffraction intensity of 1D magnetic PhCs is constantly strong because of the giant difference of reflective index between magnetic colloidal particles and surrounding solvents.^76^, ^77^ However, to ensure the balance between magnetic forces and interparticle forces, the spacing and sizes of particles tend to range from a few nanometers to a few hundred nanometers; thus the corresponding wavelength of the resultant PhCs could not cover the whole visible light spectrum. For instance, Yin and his colleagues reported a facile approach to manufacturing 1D photonic nanochains combined with sol‐gel process, as shown in Figure 4A–C.^78^ Initially, superparamagnetic colloidal nanoclusters assembled as chains under an external magnetic field, which were further coated with an additional silica layer to mechanically retain connecting in the long term. The interparticle spacing could be optimized by tuning the exposure time under a magnetic field. The critical point is to induce the stable chaining process during the sol‐gel coating due to the weak connection between neighboring nanoparticles. The resultant photonic chains dispersed randomly in solutions without magnetism and arranged into periodic alignments upon bringing in an external magnetic field with the instant appearance of diffraction colors.

**FIGURE 5:**
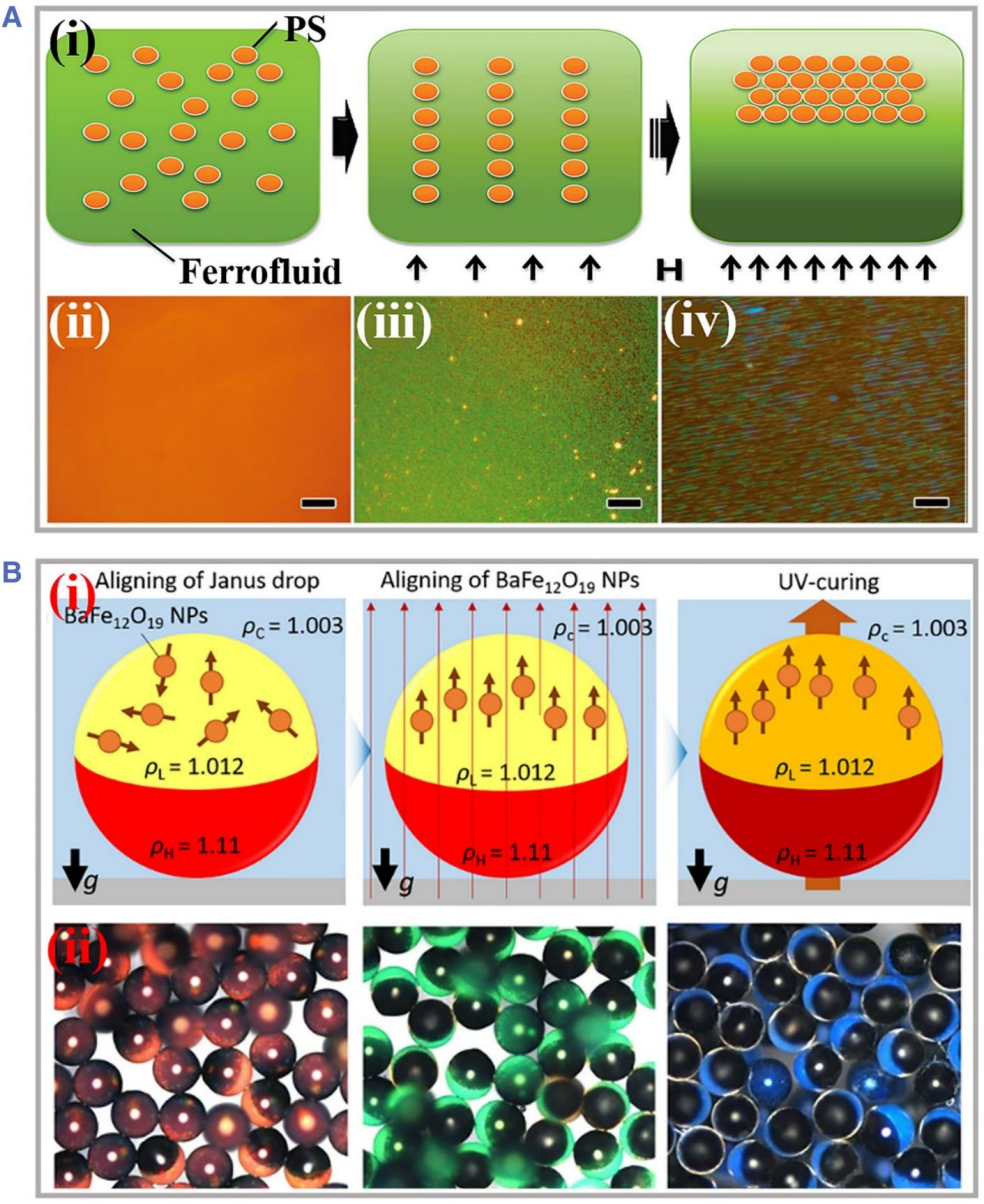
The 2D and 3D assembly of magnetic photonic crystals. (A) (i) Scheme of assembling nonmagnetic polymer beads into photonic crystal structures from 1D chains to 3D assemblies. (ii–iv) Self‐assembly process of nonmagnetic nanoparticles within a thick 2D film varying with increasing magnetic intensity. Reproduced with permission.^82^ Copyright 2010, American Chemical Society. (B) (i) Schemes of the fabrication process of Janus photonic balls, including spontaneous alignment of a drop bearing obvious inner boundary, assembly of magnetite nanoparticles under external field, and instance photopolymerization of the drop to fix Janus structures. (ii) Optical images of photonic Janus balls with red, green, and blue reflection colors. Reproduced with permission.^83^ Copyright 2020, American Chemical Society.

If the magnetic intensity is continuously increased, the dipole–dipole interactions would also be enhanced and the initial thermodynamical stability would be disturbed. The 1D chains primarily separated would aggregate in a side‐by‐side manner and gradually form 2D planar structures.^79^, ^80^, ^81^ However, these 2D planar structures are thought of as an unstable transition stage between 1D chain‐like structures and 3D colloidal crystals because these collapse processes are normally rapid along with the growth of particle concentrations and magnetic intensity. To demonstrate this evolution process, the particles would be induced to assemble with extremely crystalline structures finally. As presented in Figure 5A, Yin's group designed a rapid formation procedure by assembling nonmagnetic colloidal nanoparticles into PhCs within ferrofluids.^82^ To enhance the magnetic dipole moment of nonmagnetic particles, they adopted ferrofluids bearing elevated surface‐charge to overcome the aggregation behaviors under a strong magnetic field, and stable assembly of nonmagnetic particles from 1D chains to 3D crystallization was achieved by tuning the interplay of the magnetic dipole force and packing force. The obvious evolution processes were optically observed with the variations of magnetic intensity and volume fractions. The apparent evolution is observed optically with variations in magnetic field strength and volume fraction. On the one hand, magnetic colloidal particles with increased concentration are further localized under a strong external magnetic field and undergo crystallization processes. On the other hand, by tuning the magnetic field strength, the dipole–dipole interaction can be precisely controlled to induce particles to form 3D colloidal crystals with a non‐closed packing structure.^84^, ^85^, ^86^, ^87^, ^88^ Besides, the boundary conditions could not be ignored if the assembly processes occur in a confined space. For example, the geometry of the boundary inevitably affects the assembly behavior. The curvature of the spherical boundary will hinder the initially continuous alignment of the particles, and eventually, there will be a wavelength shift of the PhCs in certain regions. Inspired by the well‐confined assembly strategy, Kim et al. fabricated magnetic photonic Janus balls bearing controllable magnetic moments for anti‐counterfeiting tags (Figure 5B).^83^ The magnetic nanoparticles were assigned as 3D colloidal crystals in vertically aligned microfluidic drops, where the internal compartment of droplets caused by density asymmetry contributed to the colloidal crystallization. Taking advantage of the rapid polymerization of photocurable resins, the crystalline arrays were fixed stably. The different density distributions of the two components finally led to Janus structures of photonic balls. By manipulating the direction of the external magnetic field, such Janus photonic balls were applied for simultaneous programmable structural color switching.

In general, on the basis of the manipulation on the properties of magnetic field and particle concentrations, the magnetic assembly of colloidal particles into PhC structures could be successfully obtained, and the optical properties of the resultant magnetic PhCs are tunable to satisfy the practical requirements. The critical point lays on remaining the equilibrium balance between magnetic forces (dipole–dipole force and packing force) and interparticle interactions. Parameters such as charge density, polarity, pH value of the surrounding medium should be taken into consideration to retain thermodynamical stability.

## 3 BIOMEDICAL APPLICATIONS OF MAGNETIC PhCs

As mentioned above, the structural colors are derived from the inherent periodic lattices of PhCs. Compared with traditional coloration pigments or chemical dyes, the unique structural colors bearing the characteristic wavelength are chemically stable and free from bleaching. In brief, such structure‐dependent colors could be externally customized by altering microstructures or tuning diffraction properties of component materials. Astonishingly, on the basis of convenient magnetic manipulation and extensive selection of magnetic materials, magnetic PhCs have shown great superiority on brilliant structural colors bearing wide visible light band and flexible magnetism‐induced optical properties. Magnetic PhCs have been widely utilized for biomedical applications, such as novel inks for bioprinting, sensing, magnetism pigments, and so on.^89^, ^90^, ^91^, ^92^, ^93^

### 3.1 Photonic inks

**FIGURE 6:**
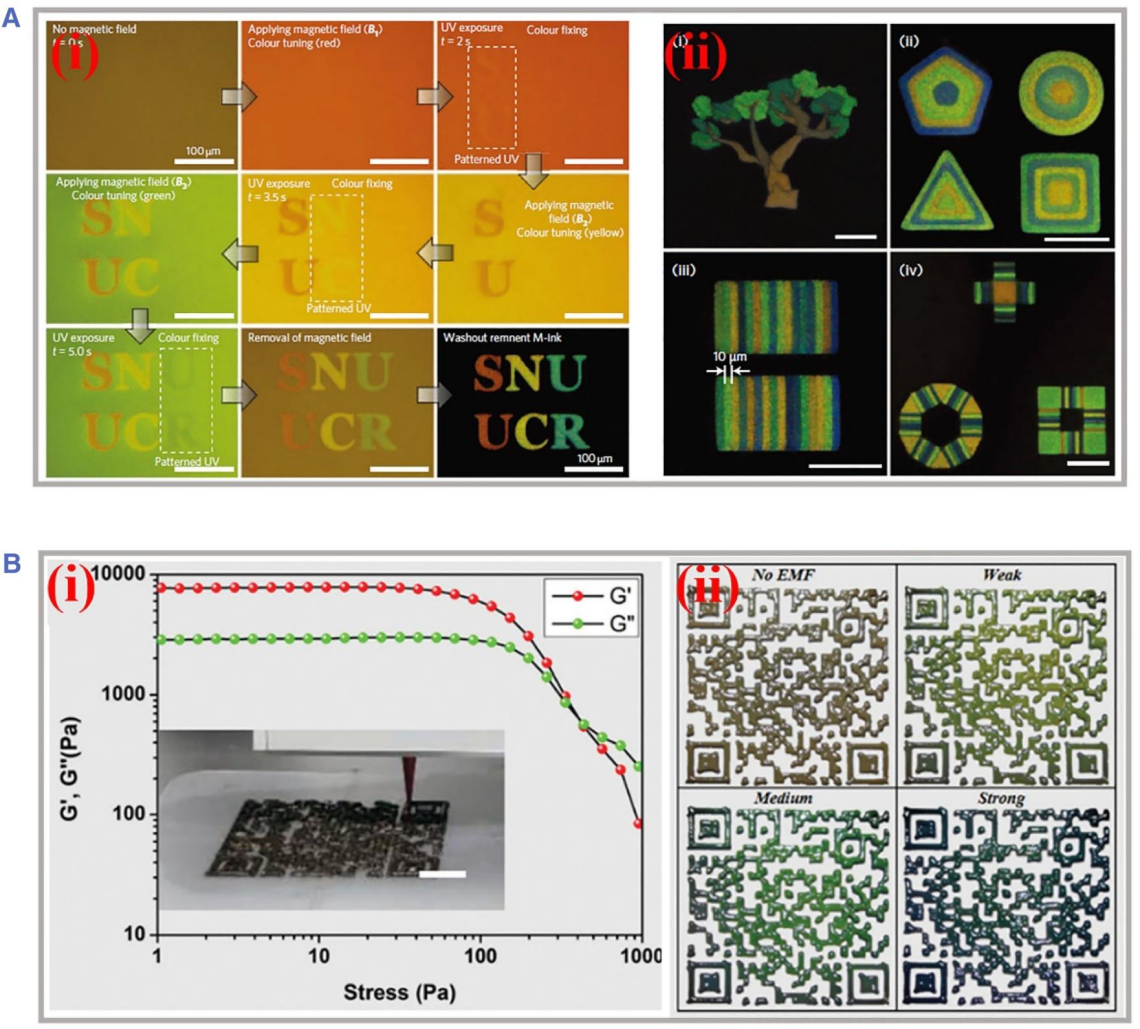
Applications of magnetic photonic crystals as printing inks. (A) (i) Rapid production of high‐resolution multiple structural colors within seconds based on superparamagnetic crystal nanoclusters dispersed in photocurable resins. (ii) Diverse patterns printed with different color compartments. Reproduced with permission.^94^ Copyright 2009, Nature Publishing Group. (B) (i) The fundamental rheological curve of the magnetite nanoparticle emulsion‐based inks. (ii) Optical images of the printed quick response (QR) codes possessing gray color initially and blue‐shift structural color with the increasing external magnetic intensity. Reproduced with permission.^95^ Copyright 2021, Royal Society of Chemistry.

By tuning the different sizes of colloids, PhCs bearing multiple brilliant colors could be obtained and act as inks for color display in a large area. However, the assembly process of PhCs with a single color is constantly time‐consuming for better crystallization. For cost‐effective manufacturing of multicolored PhCs on the same substrate simultaneously, instantaneous crystallization response to external manipulation is necessary to realize color variations. For instance, Kwon and his colleagues proposed a novel ink composed of magnetically tunable and lithographically fixable PhCs for rapid printing of structural colors, as shown in Figure 6A.^94^ The nanostructures of inks were successfully customized by external magnetism, and instantly immobilized in a photocurable resin network. Interestingly, they realized multicolored patterning within seconds on the basis of a single material. Taking advantage of the precise performance of ultra‐visible light‐induced polymerization on a well‐defined region under optimized magnetism, the superparamagnetic colloidal particles appeared different on alignment of magnetic dipole moments and the resultant scattered light varied. Furthermore, the resolution of printing was improved via four‐bit reflection intensity modulation and mixing different single‐color‐dots. Such a magnetism‐assisted printing procedure extended the practical applications of PhCs involving information anti‐counterfeiting, forgery protection, scalable multiple structure colors printing, etc.

Based on the 1D chain‐like magnetic PhCs, Yin et al. designed a magnetically rewritable photonic ink containing superparamagnetic Fe_3_O_4_ colloidal crystal clusters.^96^ The selected dispersant solvent possessed high viscosity, where the alignment of photonic nanochains could be temporarily maintained after removal of magnetism. They successfully achieved the state switch (“Off” to “On”) of photonic inks by switching on the external magnetic field. In addition, the length of photonic nanochains also influenced the lasting time of stable periodic alignments due to the equilibrium between rotational movements of nanochains and suppression of surrounding viscosities. Subsequently, by optimizing such an equilibrium state, they adopted a magnet with pre‐designed patterns to obtain a complex alignment of nanochains and realized direct writing of letters on the display substrate. Interestingly, two types of photonic inks bearing different chain lengths were uniformly mixed and magnetically assembled. After removal of the magnet, the ink presented an obvious color animation, which could be further developed for magnetic anti‐counterfeiting or color signage applications.

3D printing, an emerging additive manufacturing technology, has cast a new light on the fabrication of diverse and elaborate structures with versatile materials. The critical point for successful 3D printing is to choose appropriate inks with incredible properties. As an ideal alternative for 3D printing, photonic inks have attracted increasing attention owing to its bright and vivid structural colors. For example, Wang and his coworkers prepared magneto‐sensitive photonic inks for rapid 3D printing (Figure 6B).^95^ To better reserve the iridescent colors during the printing process, they encapsulated magnetite nanoparticle clusters into water‐in‐oil droplets, and the integral prepolymer served as final inks with excellent emulsion stability, thixotropy, and moderate viscosity. Finally, a butterfly‐shaped pattern bearing a quick response to external magnetic field intensity was printed through a direct ink writing process. By tuning the magnetic field intensity, the distances between neighboring lattice units within polymer networks underwent respective changes; thus the refractive light varied from blue to yellow according to the above mentioned Bragg's law. Different from typical aqueous‐dispersed magnetic PhCs, such inks composed of water‐in‐oil emulsion provided droplet‐confined sites for the stable assembly of magnetite particles. To some extent, this strategy partially eliminated the interference arising from the heterogeneous experimental magnetic field and prepared magnetic PhCs with better encapsulation safety and compatibility for biomedical applications.

### 3.2 Sensing

**FIGURE 7:**
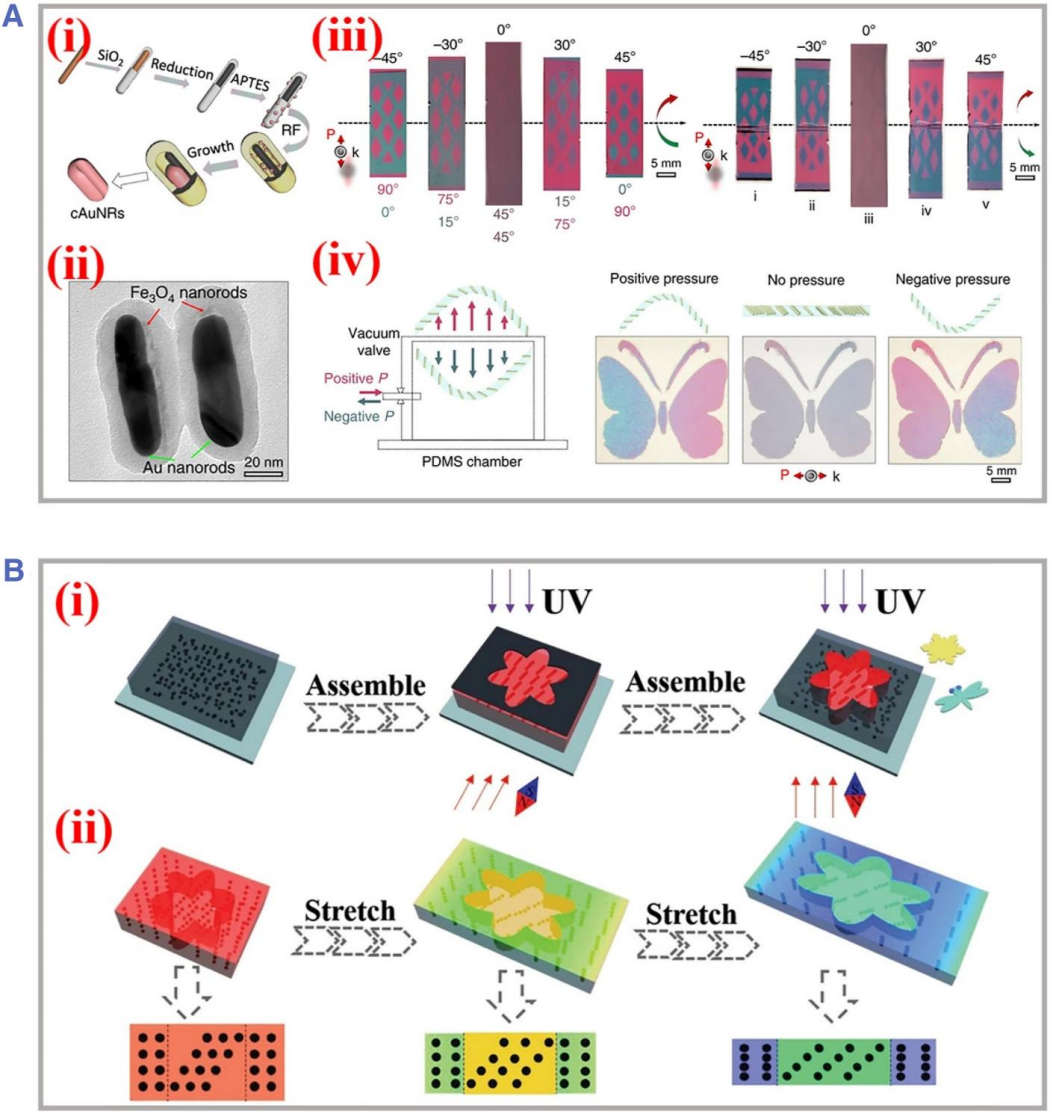
Applications of magnetic PhCs as sensory units. (A) The magnetic PhCs‐based mechanochromic device. (i–ii) The scheme and images of the well‐confined synthesis of magnetic–plasmonic hybrid nanorods. The sensory applications based on the structural colors variation including (iii) rotation and bending motions and (iv) positive and negative pressure shifts. Reproduced under terms of the CC‐BY license.^98^ Copyright 2020, The Authors, published by Springer Nature. (B) (i) Schematic manufacturing process of super‐elastic hydrogels containing induced self‐assembled magnetic nanoparticles. Ultraviolet light polymerization was used to instantly fix the structural color. (ii) The sensory mechanism based on the color change during the stretching process. The stretching changed the primary alignments of nanoparticles and led to variation of reflection lights. Reproduced with permission.^97^ Copyright 2019, WILEY‐VCH Verlag GmbH & Co. KGaA, Weinheim. PhCs, photonic crystals.

Recently, optical sensors have been widely integrated in the field of biomedical engineering with the merits of indirect contact, rapid responsiveness, and minimum interference. As one representative type of optical sensors, PhCs‐based sensory strategy targets the intrinsic refractive signals, which are generated owing to variations of the refractive index or reflection wavelengths, and subsequently propagated by the fiber optics waveguide. Based on the manipulation of photonic properties performed by an external magnet, magnetic PhCs have been considered as promising functional units for the sensitive measurement of diverse signals. For instance, Yin's group reported mechanochromic films composed of magnetic plasmonic hybrid nanorods bearing programmable colorimetric responses, as illustrated in Figure 7A.^98^ First, these hybrid nanorods (Fe_3_O_4_/Au) were synthesized via seed‐mediated growth confined within polymer shells. Then, such nanorods were incorporated into polymer films, where the orientations were well controlled by convenient magnetic alignments. Based on the internal asymmetric anisotropic alignment, machanochromic devices with designed patterns were fabricated and applied for optical sensing of both linear and angular motions using easy magnetic manners. It was demonstrated that such reversible magnetic interactions could realize the efficient assembly of colloidal nanoparticles and thus, broadened the manufacturing procedures of soft actuators and robots, featured with simultaneous sensing of dual/multiple signal variations for wider biomedical applications.

Apart from the magnetite colloidal nanoparticles, the polymer matrix as a dispersion agent is another critical component of magnetic PhCs. The periodic alignments of colloidal nanoparticles are not only determined by the intensity or orientation of the external magnetic field but also influenced by the properties of the surrounding medium, such as polarity and viscosity. Astonishingly, by integrating functional components within polymer networks, the magnetic PhCs could be attached with more responsiveness to external stimuli and enrich the sensory parameters. For example, Zhao and his team designed super‐elastic hydrogels embedded with magnetic responsive PhCs through a two‐step facile synthesis procedure, as shown in Figure 7B.^97^ The core‐shell magnetic colloidal nanoparticles were first induced to self‐assemble as an orderly packing alignment, and then super‐elastic hydrogels were polymerized in situ to fix the photonic nanostructures. It was found that the hybrid possessed great resistance to interference of ions. By tuning the orientation and intensity of the external magnet, the ordered lattice of self‐assembly units would not collapse, and thus, regional magnetic‐induced polymerization could be realized with pre‐designed patterns. The lattice constant would change during the stretching process and the respective structural colors would vary from blue to red. Moreover, such color conversions were recyclable with excellent stability. These features endowed this super‐elastic hydrogel film potential for stress–strain and shape deformation sensing.

Similarly, Ge et al. established a magnetic PhCs‐based polymer film for relative humidity sensing.^99^ Typically, a thin film composed of superparamagnetic Fe_3_O_4_@SiO_2_ colloids were deduced into 1D chain‐like structures under magnetism, and then, instant photo‐polymerization of hygroscopic poly(ethylene glycol) methacrylate and poly(ethylene glycol) diacrylate happened to fix photonic structures. The liquid precursor was optically transparent, which would not disturb or weaken the periodic nanostructures‐derived structural colors. Most importantly, such a polymer matrix strongly absorbed water within humid air and swelled rapidly, leading to the variation of the average refractive index. Besides, the sensitivity and speed of responsiveness could be manually optimized by tuning the cross‐linking level and matrix thickness of the composite film to meet different needs of practical applications. The relationship between shift of wavelength and air humidity was established as a precise sensory principle. The merits such as low cost and less power consumption, together with environment compatibility, demonstrated the feasibility of integrating diverse functional polymers into magnetic PhCs for individual sensing of a certain index.

Definitely, there still exist abundant applications of magnetic PhC materials, such as anti‐counterfeiting, multiple encoding, light guide fibers, coloration pigments, and so on. In brief, on the basis of easy magnetic manipulation and excellent optical responsiveness, the magnetic PhCs have shown great potential in the field of biomedical engineering as a reliable tool for magnetic resonance imaging (MRI) auxiliary diagnosis, magnetothermal effect, magnetoelectricity, etc.

## 4 CONCLUSION AND PERSPECTIVE

In this review, we have summarized fundamental magnetic self‐assembly principles of PhCs including 1D chain, 2D plane, and 3D colloidal crystals. Bearing merits such as convenient manipulations, lower fabrication costs, and higher production efficiency, magnetic PhCs materials have been widely integrated for biomedical applications such as printing inks, sensing, color display, and so on. The instance responsiveness of tunable and extremely reversible structural colors under an external magnetic field could also be further incorporated within a new colloidal assembly strategy for other nonmagnetic or polymer nanoparticles. It was worth mentioning that these superior features prepare magnetic PhC materials for diverse applications, especially in the field of industry, where the low cost and easy operations broadened the mass production for further commercialization.

Although diverse magnetic PhCs with desirable functions have been designed and synthesized, a huge gap still remains between laboratory and industrial applications. For instance, the assembly of simple magnetic 1D chain PhCs was dominated by the surface charge of nanoparticles, which contributed to the balance between dipole–dipole attractive forces and repulsive forces. Under certain practical conditions, the complex properties of surrounding solutions would inevitably weaken the repulsive forces, influence the above balances, and hinder the assembly. Therefore, necessary chemical modifications of nanoparticles should be involved to induce repulsive forces during synthesis procedures. Moreover, the limited sample size of magnetic PhCs also constricted the formation of large‐area highly ordered structures due to the long‐time consumption. It still faces challenges to maintain the stable PhC structures with high quality during the long‐term manufacturing process. In addition, the widely used magnetic nanoparticles, such as Fe_3_O_4_, possess strong optical absorption, which weakens the intrinsic structural colors of PhCs to some extent, and oxidation reactions inevitably lead to brittle periodic alignments. Thus, there is still a long way to go for magnetic PhCs to be used in practical applications.

In conclusion, magnetic PhC materials have actually obtained tremendous advancements as coloration pigments, printing inks, sensory units for active color display, anti‐counterfeiting, encoding, etc. Besides, magnetic PhCs have been considered a powerful contrast agent in the field of MRI due to their tunable sizes, flexible paramagnetism, and excellent biocompatibility. In addition, magnetothermal effects of magnetic PhCs are also important tools for tumor thermotherapy in clinical applications. Future explorations and focus should be on the exploration of new building blocks with diverse morphologies and magnetic assembly mechanism with excellent instance and stability, and more advanced materials should be integrated to impart magnetic PhCs with programmable functionalities. Last but not least, to widen the potentials of magnetic PhCs for biomedical applications, the interactions on the biological interface should be taken into consideration, such as the compatibility of components, flexible adaptation, safety, etc. We expect this comprehensive review will give birth to new sparks for more breakthrough of magnetic PhCs materials.

## AUTHOR CONTRIBUTIONS

Jinglin Wang conceived the idea; Hanxu Chen wrote the manuscript and edited the figure; Hanxu Chen and Ning Li revised the manuscript; Zhuxiao Gu and Hongcheng Gu contributed to the manuscript preparing.

## CONFLICTS OF INTEREST STATEMENT

There are no conflicts to declare.

## ETHICS STATEMENT

This review does not include human or animal research.
